# The reliability, validity and factorial structure of the Swahili version of the 7-item generalized anxiety disorder scale (GAD-7) among adults living with HIV from Kilifi, Kenya

**DOI:** 10.1186/s12991-020-00312-4

**Published:** 2020-10-28

**Authors:** Moses K. Nyongesa, Paul Mwangi, Hans M. Koot, Pim Cuijpers, Charles R. J. C. Newton, Amina Abubakar

**Affiliations:** 1 Neuroassessment Group, KEMRI/Wellcome Trust Research Programme, Centre for Geographic Medicine Research (Coast), KEMRI, Box 230, Kilifi, Kenya; 2 Department of Clinical, Neuro- and Developmental Psychology, Amsterdam Public Health Research Institute, Vrije Universiteit Amsterdam, Amsterdam, Netherlands; 3 Department of Public Health, Pwani University, Kilifi, Kenya; 4 Department of Psychiatry, University of Oxford, Oxford, UK; 5 Institute for Human Development, Aga Khan University, Nairobi, Kenya

**Keywords:** Psychometric properties, Factor analysis, Swahili GAD-7, HIV/AIDS, Adults, Kenya

## Abstract

**Background:**

Generalized Anxiety Disorder (GAD) is under-investigated in people living with HIV/AIDS from sub-Saharan Africa. In part, this is due to paucity of culturally appropriate measures for GAD which are psychometrically robust. This study aimed to evaluate the reliability, factorial structure, and validity of Swahili version of the 7-item GAD questionnaire (GAD-7) among adults living with HIV.

**Study design:**

Descriptive cross-sectional study.

**Methods:**

450 adults receiving comprehensive care from an HIV specialized clinic in Kilifi County, coastal Kenya, were consecutively recruited. Swahili versions of GAD-7, Patient Health Questionnaire (PHQ-9) and a 12-item HIV stigma scale were administered alongside measures of psychosocial and health-related characteristics. Internal consistency, test–retest reliability, factorial structure, convergent validity, and discriminant validity of Swahili GAD-7 were examined using Cronbach’s alpha (*α*), intra-class correlation coefficient (ICC), Confirmatory Factor Analysis (CFA), Pearson’s correlation, and analysis of covariance (ANCOVA), respectively.

**Results:**

Internal consistency of Swahili GAD-7 was good, *α* = *0.82 (95% *CI* 0.78, 0.85)*. Its test–retest reliability (2 weeks apart) was acceptable, ICC = *0.70 (95% *CI* 0.55, 0.81*). A confirmatory analysis of a one-factor solution indicated an excellent fit to the hypothesized structure (RMSEA = 0.00 [95% confidence interval 0.00, 0.05], CFI = 1.00, TLI = 1.00). Multi-group CFA substantiated factorial invariance for sex and age for the one-factor structure of Swahili GAD-7. Scores of GAD-7, Swahili version, significantly correlated with those of PHQ-9 (*r* = *0.73; p* < *0.001)* and the HIV stigma scale (*r* = *0.36; p* < *0.001)* suggesting good convergent validity. Statistically significant differences were observed between participants on first-line antiretroviral therapy compared to those on second-line treatment *(F [1, 441]* = *5.55, p* = *0.02)* indicative of good discriminant validity of Swahili GAD-7*.*

**Conclusion:**

GAD-7 Swahili version retained its original unidimensional latent structure with good psychometric properties among adults living with HIV from Kilifi, Kenya. It can be used to identify symptoms of GAD in similar research settings. However, to confidently identify those in need of mental health treatment or referral services in HIV primary care clinics, more research on the validity of Swahili GAD-7 is needed especially its discriminant validity and diagnostic accuracy at different cut-off scores.

## Introduction

Generalized anxiety disorder (GAD) is one of the most common mental disorders and a significant contributor to the global burden of disease [1]. It is characterized by excessive anxiety and worry about events or activities that are difficult to control [2]. In the general population, the estimated lifetime prevalence of GAD ranges from 0.4 to 5.7% [3]. In primary healthcare (PHC) settings, prevalence of GAD symptoms ranges from 3.7 to 14.8% [4], and GAD accounts for over half of anxiety disorders seen in these settings [5]. Among people living with HIV/AIDS (PLWHA) attending primary care clinics, high prevalence of GAD symptoms has been reported from high-income settings at 23% [6] and over 30% [7, 8] from low- and middle-income countries (LMICs). This high prevalence of GAD symptoms in PLWHA may result from reaction to initial diagnosis of HIV or recurring and escalating anxiety symptoms in response to disease progression.

Generalized Anxiety Disorder is a debilitating condition. It significantly impairs an individual’s health-related quality of life [9, 10], and it influences self-care, help seeking behaviour, and interpersonal functioning (family and social) [11, 12]. GAD increases propensity for adverse health behaviour such as smoking and sedentary lifestyle [13] and is strongly associated with comorbid psychiatric disorders, major depressive disorder being the commonest [14]. Among PLWHA, GAD is a risk factor for non-adherence to antiretroviral therapy [15]. The economic burden of GAD in terms of lost work productivity (indirect costs) and costs of medical services (direct costs) has also been found to be considerable [16, 17].

Despite being highly prevalent among PLWHA and impairing, in comparison to other mental disorders, GAD has received less attention with respect to media, research and public health efforts [4, 13, 18]. Furthermore, this emotional disorder remains under-diagnosed and under-treated in primary care settings of most countries [13, 19]. Previous research has revealed general challenges to the identification and treatment of disabling mental disorders in PHC settings of LMICs. These include: a lack of time in busy PHC settings [20], inadequate knowledge related to symptoms and management of mental illnesses among PHC staff [20, 21], poor integration of mental health services in PHC [20, 22] and scarcity of specialized mental health personnel [21, 23].

In part, the inattention to GAD in LMICs may also be due to the paucity of culturally sensitive and psychometrically robust measures [24]. The 7-item GAD scale (GAD-7), which was developed in a Western setting, screens and measures severity of GAD [18], but has limited validation in LMICs. For instance, in sub-Saharan Africa (SSA), to the best of our knowledge, only one study has attempted to validate a local version of GAD-7 (Shona version) for use in PHC settings in Zimbabwe [24], although not exclusively among PLWHA. This local version of GAD-7 (at an optimal cutoff ≥ 10) showed good performance characteristics, i.e., Cronbach alpha of 0.87, with sensitivity of 89% (95% confidence interval: 81–94%) and specificity of 73% (95% confidence interval: 65–80%) against the Structured Clinical Interview of the Diagnostic and Statistical Manual of Mental Disorders-IV (SCID), a gold standard tool for the diagnosis of GAD.

In view of availing more locally validated GAD measures in LMICs, specifically SSA where both GAD and HIV are highly prevalent [7, 8, 25], this study evaluated the psychometric properties of a Swahili version of GAD-7 among adults living with HIV from rural Kilifi, Kenya. Specific aims were to describe: (i) the internal consistency; (ii) test–retest reliability; (iii) factorial structure; (iv) convergent validity; and (v) discriminant validity of Swahili GAD-7.

## Methods

### Study setting and participants

This work is part of data collected from a larger cross-sectional study looking at different outcomes in PLWHA, including health-related quality of life and mental health. The cross-sectional study was conducted at the Center for Geographic Medicine Research (CGMR), located in Kilifi County, between 20th February and 15th April 2018. The centre is within Kilifi County Hospital, the main referral hospital. In Kilifi County, prevalence of HIV in adults is estimated as 4.5% [26]. Participants were adults living with HIV attending an HIV specialized clinic at the Kilifi County Hospital. To be included in the study, they had to be 18 to 60 years old, with confirmed HIV-positive status, on antiretroviral therapy, and able to comprehend and/or communicate in the national language (Kiswahili) since this language was used during administration of all study instruments and asking for consent. We did not include elderly individuals (above 60 years) living with HIV because of the increased likelihood of illnesses associated with advancing age, which may have an impact on quality of life [27], an outcome of interest in the larger study. In total, 512 participants visiting the HIV clinic for their appointments were approached to participate, 44 of whom declined participation, whereas 18 were excluded because they were over 60 years of age (*n* = 11) or could not comprehend or communicate in Kiswahili (*n* = 7). The remaining 450 adults living with HIV participated in the study.

### Study instruments

#### Sociodemographic and health-related characteristics

In the larger study, sociodemographic, health and treatment history forms were administered in a face-to-face interview in private rooms at the HIV clinic. We only present variables relevant to the present analyses. These include sociodemographic data on age, sex, marital status, level of education, employment, and information about participant antiretroviral regimen, World Health Organization (WHO) disease staging [28], and presence of any current chronic medical illness (they have been made aware of by their clinician).

#### Clinical history

A clinical record form was used to extract the following data from the medical records of recruited participants at the HIV clinic: antiretroviral regimen (first line versus second line), viral load (within the last one year), cluster of differentiation 4 [CD4] cell count, and WHO-based disease staging. However, we found no recent follow-up CD4 tests for all the recruited participants during data extraction. The available CD4 cell count information, not for all participants, was that when they were being enrolled into care. From the medical records, there were major missing data (*n* = 145) for the most recent viral load tests of our participants. Therefore, from the clinical data, we only present information on antiretroviral regimen (first line versus second line) and disease staging.

#### 7-Item generalized anxiety disorder scale (GAD-7) [18]

GAD-7, Swahili version, was administered to all study participants. Using a Likert scale of 0 (not at all) to 3 (nearly every day), participants are asked how often they have been bothered by problems such as feeling nervous, worrying, or restlessness in the past two weeks. Total score ranges from 0 to 21. According to the manual, summated scores of 5–9 indicates mild GAD symptoms, 10–14 indicate moderate GAD symptoms, and a summated score ≥ 15 indicates severe GAD symptoms. Previous research has suggested that the GAD-7 is a valid screening tool for GAD and for assessing its severity in clinical practice and research [18].

#### 9-Item patient health questionnaire (PHQ-9) [29]

PHQ-9 screens and provides a measure of severity of depressive symptoms. It is scored on a Likert scale of 0 (not at all) to 3 (nearly every day).The summated score ranges between 0 and 27, with a cut-off of 10 generally accepted as a positive screen for depressive symptoms [30]. Used among PLWHA in Kenya [31], this measure presented good internal consistency (Cronbach’s α = 0.78) and acceptable test–retest reliability (intra-class correlation coefficient [ICC] = 0.59). In this study, its internal consistency was good, *α* = 0.81 (95% CI 0.78, 0.84).

#### 12-Item HIV stigma scale [32]

Was used to assess participant perceived HIV-related stigma under four dimensions of: (i) personalized stigma; (ii) disclosure concerns; (iii) negative self-image; and (iv) concerns with public attitudes. Items on this scale are rated as 1 (strongly disagree), 2 (disagree), 3 (agree), and 4 (strongly agree). Total score ranges between 12 and 48, higher scores indicative of greater level of perceived HIV-related stigma. In the initial validation conducted in Sweden, Cronbach’s alpha was > 0.70 [32]. In this study Cronbach’s alpha was 0.81 (95% CI 0.78, 0.83).

### Translations

In line with international guidelines for translation of tools in health research (https://www.who.int/substance_abuse/research_tools/translation/en/), all questionnaires were independently translated from English to Swahili by two staff members fluent in both languages. Back-translations into English were then done by another independent pair of translators. To ensure content, conceptual, semantic and idiomatic equivalence of the questionnaires [33], a committee of HIV researchers (natives of Kenya, knowledgeable about the Kenyan culture, bilingual and fluent in both English and Kiswahili) and the translators then held a harmonization meeting. Discrepancies in the translations were resolved by consensus. The final versions were obtained following incorporation of changes resulting from pretesting procedures.

### Statistical analysis

Cronbach’s alpha was used to evaluate internal consistency of Swahili GAD-7. ICC assessed test–retest reliability (using data of 60 participants rescreened 2 weeks following initial assessment). Using structural equation modelling, confirmatory factor analysis (CFA) with diagonally weighted least squares was used to determine the factor structure of Swahili GAD-7. An a priori model was specified, where one factor underlies the item responses and each item loads onto this unidimensional factor. The loadings associated with responses from the Swahili GAD-7 were then generated. The choice to confirm a one-factor structure was informed through an exploratory factor analysis (EFA) that checked for a possibility of the posited two-dimensional structure of GAD-7 [34, 35]. We examined the goodness-of-fit between the hypothesized model and the data using the following fit indices: root mean square error of approximation [RMSEA], comparative fit index [CFI] and Tucker–Lewis index [TLI]). Using multiple indices provides a conservative and reliable evaluation of model fitness [36]. Based upon the literature [37–39], fit indices were considered acceptable if RMSEA ≤ 0.08, CFI and TLI ≥ 0.90, or excellent if RMSEA ≤ 0.06, CFI and TLI ≥ 0.95. Prior to CFA, we checked for sampling adequacy using Kaiser–Meyer–Olkin (KMO) value and Bartlett’s test of sphericity. We also examined inter-item correlations using Kendall tau-b correlation (in view of the ordinal measurement level of the items of GAD-7). Multi-group CFA was conducted to determine measurement invariance (configural, metric, and scalar invariance) between sexes (female versus male) and age groups (young adults 18–35 years versus middle-aged adults 36–60 years) for the hypothesized unidimensional factor structure of Swahili GAD-7. We used difference in CFI (ΔCFI), cut-off value ≤ 0.01, to define invariance as this is the most widely used and empirically best supported criterion [40–42]. Convergent validity was assessed through correlation of GAD-7 scores with scores of both PHQ-9 and 12-item HIV stigma scale (Pearson’s product correlation). For discriminant validity, independent Student’s t-test followed by analysis of covariance (ANCOVA) were used to evaluate whether GAD-7 is sensitive to antiretroviral regimen (first-line versus second-line treatment). We used antiretroviral regimen as a proxy for perceived treatment success (switching to second-line treatment perceived as poor treatment success and expected to be related to higher GAD-7 scores) because of missing data on biological markers of disease progression (viral load or CD4 cell count) from the participant clinical records. All analyses were conducted using STATA (version 14.0) statistical software package except test–retest and CFA analyses which were done on R (version 3.4.1) software.

## Results

### Participant description

Table 1 summarizes sociodemographic, health and treatment characteristics of study participants by sex. Majority of the study participants were females (79.1%). The mean age of the 450 recruited participants was *42.7 years (Standard Deviation *[SD] = *9.7)*. Participants’ median PHQ-9 score was *3 (Inter-Quartile Range *[IQR]:* 1—7)*. For perceived HIV-related stigma, mean score was *28.4 *(SD = *7.7*).

**Table 1:** Study participants’ sociodemographic, health, and treatment characteristics by gender

Characteristic	Total sample *N* = 450	Female *n* = 356	Male *n* = 94
Age group			
18–24 years	20 (4.4%)	11 (3.1%)	9 (9.6%)
25–35 years	87 (19.3%)	79 (22.2%)	8 (8.5%)
36–49 years	223 (49.6%)	175 (49.2%)	48 (51.1%)
50–60 years	120 (26.7%)	91 (25.6%)	29 (30.9%)
Marital status			
Married/cohabiting	196 (43.6%)	142 (39.9%)	54 (57.5%)
Separated/divorced/widowed	197 (43.8%)	173 (48.6%)	24 (25.5%)
Never married	57 (12.7%)	41 (11.5%)	16 (17.0%)
Education			
Tertiary	22 (4.9%)	13 (3.7%)	9 (9.6%)
Secondary	66 (14.7%)	42 (11.8%)	24 (25.5%)
Primary	239 (53.1%)	180 (50.6%)	59 (62.8%)
None	123 (27.3%)	121 (34.0%)	2 (2.1%)
Employment			
Formally employed	53 (11.8%)	34 (9.6%)	19 (20.2%)
Self-employed	117 (26.0%)	95 (26.7%)	22 (23.4%)
Unemployed	269 (59.8%)	219 (61.5%)	50 (53.2%)
Other	11 (2.4%)	8 (2.3%)	3 (3.2%)
Current chronic medical illness^a^			
Present	37 (8.2%)	30 (8.4%)	7 (7.5%)
Not present	413 (91.8%)	326 (91.6%)	87 (92.6%)
Antiretroviral regimen^b^			
First line	425 (95.3%)	335 (95.2%)	90 (95.7%)
Second line	21 (4.7%)	17 (4.8%)	4 (4.3%)
WHO disease staging			
Stage 1	417 (93.7%)	334 (94.9%)	83 (89.3%)
Stage 2	22 (4.9%)	14 (4.0%)	8 (8.6%)
Stage 3	3 (0.7%)	2 (0.6%)	1 (1.1%)
Stage 4	3 (0.7%)	2 (0.6%)	1 (1.1%)

All variables had complete data except for antiretroviral regimen (4 missing observations) and WHO disease staging (5 missing observations)

^a^Based on client self-reporting as informed by their clinician

^b^All the 21 participants on second-line medication were initially started on first line

### Item characteristics

Table 2 presents the *mean (*SD*)* participant scores for each of the GAD-7 items alongside percentage response across the ordinal Likert scale options. Of important note is that responses were distributed across all the Likert options in the expected direction indicative of no major ceiling or floor effects.

**Table 2:** Swahili GAD-7 item descriptive and CFA factor loadings

Item code	Item description^a^	Item descriptive					
		Item score^b^	% responses in each category				Factor loadings^c^
			0	1	2	3	
GAD1	Feeling nervous, anxious or on edge	0.36 (0.65)	71.3	22.9	3.8	2.0	0.76
GAD2	Not being able to stop or control worrying	0.24 (0.57)	81.3	14.0	3.6	1.1	0.74
GAD3	Worrying too much about different things	0.39 (0.73)	71.7	20.9	3.8	3.6	0.77
GAD4	Trouble relaxing	0.43 (0.80)	71.8	18.4	4.9	4.9	0.53
GAD5	Being so restless that it is hard to sit still	0.30 (0.65)	78.0	16.9	2.5	2.7	0.47
GAD6	Becoming easily annoyed or irritable	0.61 (0.83)	55.4	33.7	5.4	5.6	0.51
GAD7	Feeling afraid as if something awful might happen	0.33 (0.60)	72.4	23.6	2.4	1.6	0.66
	GAD-7 Total item score	2.66 (3.35)	NA	NA	NA	NA	NA
	GAD-7 Total item score (median [IQR])	1 (0—4)	NA	NA	NA	NA	NA

IQR: inter-quartile range

NA: not applicable

0-not at all, 1-several days, 2-more than half the days, 3-nearly everyday

^a^Item requires respondents to consider the past 2 weeks

^b^Item score presented as mean (SD)

^c^Item loadings to the one-factor structure using confirmatory factor analysis

### Tool reliability

Cronbach alpha for the Swahili version of GAD-7 revealed very good internal consistency, *α* = *0.82 (95% confidence interval [95% *CI*] 0.78, 0.85)*. Table 3 presents item–total correlations, the correlation of each item with the rest of the items, inter-item covariance and item alphas. Test–retest reliability of Swahili GAD-7 as indicated by intra-class correlation coefficient was acceptable, ICC = *0.70 (95% *CI* 0.55, 0.81).*

**Table 3:** Item correlation, inter-item covariance and item alphas

Item	Item–test correlation	Item–rest correlation	Average inter-item covariance	Alpha^a^
GAD1	0.78	0.68	0.37	0.78
GAD2	0.76	0.65	0.38	0.79
GAD3	0.78	0.68	0.38	0.78
GAD4	0.65	0.51	0.42	0.81
GAD5	0.60	0.44	0.44	0.82
GAD6	0.61	0.46	0.43	0.82
GAD7	0.70	0.58	0.40	0.80

The displayed values are for standardized items

^a^The item alpha presented is if the respective item is deleted

### Factorial structure

**Fig. 1:**
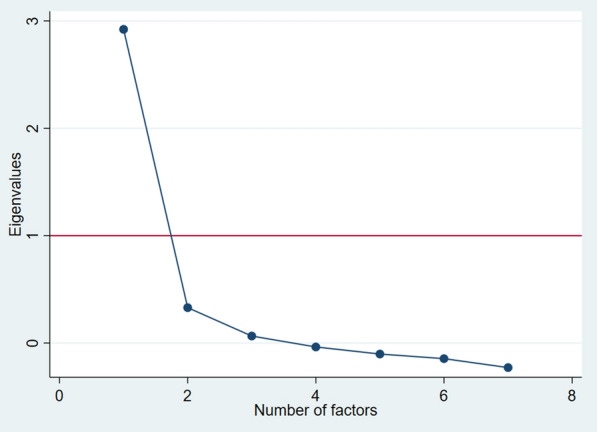
Scree plot of the eigen value

Prior to CFA, we examined KMO and Bartlett’s test of sphericity for sampling adequacy and ran an EFA to check the possibility of a two-dimensional factorial structure. KMO value was 0.84 while Bartlett’s test of sphericity was highly significant *χ*^*2*^ = *1057.32, p* < *0.001.* These results suggested that there were correlations in the dataset appropriate for factor analysis. Table 4 presents Kendall’s tau-b inter-item correlation coefficients. The correlations ranged from *r* = *0.3* to *r* = *0.7* and were all statistically significant at *p* < *0.001*. EFA analysis showed a clear one-factor structure. The first factor was the only one with an eigen value above one (2.92) and explained 81.4% of the variance. Figure 1 shows the scree plot of the eigen values which suggests a one-factor structure best fit the data.

**Table 4:** Kendall tau-b inter-item correlation coefficient

	GAD1	GAD2	GAD3	GAD4	GAD5	GAD6	GAD7
GAD1	1.00						
GAD2	0.69	1.00					
GAD3	0.54	0.60	1.00				
GAD4	0.36	0.34	0.34	1.00			
GAD5	0.29	0.35	0.34	0.50	1.00		
GAD6	0.32	0.30	0.37	0.26	0.27	1.00	
GAD7	0.44	0.40	0.40	0.33	0.26	0.36	1.00

All correlations were statistically significant at *p* < 0.001

In CFA, all the GAD-7 Swahili items loaded well on this one-factor solution (item factor loading ≥ 0.5). Table 2 presents the factor loadings. Goodness-of-fit indices for the unidimensional factorial structure of Swahili GAD-7 were all excellent (RMSEA < 0.01 [95% CI 0.00, 0.05]; CFI = 1.00; TLI = 1.00).

### Measurement invariance across sex and age groups

Multi-group CFA was used to determine the extent to which the unidimensional factorial structure of Swahili GAD-7 was invariant across sex (female versus male) and age groups (young adults versus middle-aged adults). Overall, this multi-group analysis confirmed sex and age invariance of Swahili GAD-7.

In a model assuming the same item-factor assignment across sex and age groups (configural invariance), the one-factor solution of Swahili GAD-7 fitted the data well in both groups (Table 5). This is an indication that similar latent constructs have been measured in the groups hence the one-factor measurement model is acceptable for both sexes and age groups.

**Table 5:** Goodness of fit indices for multi-group confirmatory factor analysis of unidimensional factorial structure of Swahili GAD-7

Invariance model	RMSEA^a^ (95% CI)	TLI^b^	CFI^b^	ΔCFI^c^	Reference model
Sex					
1. Configural invariance	0.000 (0.000–0.018)	1.028	1.000	–	–
2. Metric invariance	0.000 (0.000–0.038)	1.012	1.000	< 0.001	1
3. Scalar invariance	0.000 (0.000–0.031)	1.015	1.000	< 0.001	2
Age					
1. Configural invariance	0.000 (0.000–0.036)	1.017	1.000	–	–
2. Metric invariance	0.025 (0.000–0.057)	0.989	0.991	0.009	1
3. Scalar invariance	0.032 (0.000–0.059)	0.982	0.983	0.008	2

*RMSE* root mean square error of approximation, 95% CI–95% confidence interval, *TLI* Tucker–Lewis index, *CFI*-comparative fit index; Δ–change in

^a^Acceptable fit ≤ 0.08, excellent fit ≤ 0.06

^b^Acceptable fit ≥ 0.90, excellent fit ≥ 0.95

^c^Acceptable cut-off to detect invariance ≤ 0.01

Assuming same item-factor assignment but also constraining factor loadings to equivalence across sex and age groups (metric invariance), all the goodness-of-fit indices for the one-factor solution of Swahili GAD-7 were excellent (Table 5). Comparing this metric invariance model to the least stringent configural invariance model, ΔCFI was < 0.001 and 0.009 for sex and age groups, respectively.

Assuming same item-factor assignment, but also constraining factor loadings and item intercepts to equivalence across sex and age groups (scalar invariance), all goodness-of-fit indices for the one-factor solution of Swahili GAD-7 were also excellent (Table 5). Comparing this scalar invariance model to the less restrictive metric invariance model, ΔCFI was < 0.001 and 0.008 for sex and age groups, respectively.

Evaluating all the goodness-of-fit indices and ΔCFI indicated that the scalar invariance model fitted the data best for sex group while metric invariance model fitted the data best for age group.

### Convergent and discriminant validity

Scores of GAD-7, Swahili version, highly and moderately correlated with those of PHQ-9 *(r* = *0.73; p* < *0.001)* and 12-item HIV stigma scale *(r* = *0.36; p* < *0.001),* respectively. These patterns of results support convergent validity of Swahili GAD-7. Raw mean scores on Swahili version of GAD-7 were significantly higher among study participants on second-line antiretroviral treatment compared to those on first-line treatment (*4.24 versus 2.60; p* = *0.03*). Using ANCOVA adjusted for age, sex and presence of any chronic medical illness, the observed difference remained statistically significant *(F [1, 441]* = *5.55, p* = *0.02)* suggestive of good discriminant validity of Swahili GAD-7.

## Discussion

We sought to investigate the psychometric properties and factorial structure of Swahili version of GAD-7 among adults living with HIV from Kilifi. To the best of our knowledge, this is the first study from SSA that explores psychometric properties of GAD-7 exclusively among PLWHA. In summary, we found that Swahili version of GAD-7, had good internal consistency and acceptable test–retest reliability. Swahili GAD-7 retained unidimensional latent structure where all items loaded well on a one-factor solution and this model was invariant across sex and age groups. The scale also presented good convergent and discriminant validity.

The Swahili GAD-7 had Cronbach alpha of 0.82 which suggests that it is internally consistent. Rating scales are considered to have acceptable-to-excellent internal consistency if Cronbach’s alpha ranges between 0.70 and 0.95 [43]. Our finding corroborates previously reported findings of good internal consistency of GAD-7 from its original validation study [18], and other validation studies involving a general population from Germany [44], psychiatric sample from Portugal and United States [34, 45], pregnant women from Peru [46], and PHC population with high HIV burden from Zimbabwe [24].

The stability of GAD-7 between evaluations over time is rarely investigated globally with no data from SSA. Adding to the overall limited body of knowledge about test–retest reliability of GAD-7, we report that Swahili GAD-7 is stable for evaluations done 2 weeks apart in SSA context. Spitzer et al. [18] in the original validation of GAD-7, reported a high test–retest reliability of GAD-7 (ICC = 0.83). Sousa, et al. [45] investigating stability of a Portuguese version of GAD-7 for evaluations carried out 1 week apart also found that the scale had good test–retest reliability (item ICC range = 0.56 to 0.93).

This is the first study that reports on factorial structure of GAD-7 among PLWHA and specifically from SSA context. We first checked for sampling adequacy (using KMO value and Bartlett’s test of sphericity), which we found was appropriate for factor analysis. The inter-item correlation coefficients also ranged from moderate to strong and were all highly significant. We then ran EFA which showed a clear one-factor structure of the Swahili GAD-7. With this supporting background, we proceeded to conduct CFA. We found that Swahili GAD-7 retained the unidimensional latent structure. All items loaded well to a one-factor solution (factor loadings ≥ 0.5) with excellent goodness-of-fit indices (RMSEA, CFI, TLI), as recommended in the literature [36, 44]. Measurement invariance (i.e., configural, metric, and scalar) was explored across both sex and age groups. We note that majority of the study participants were females. This high recruitment of females is expected since women in SSA, compared to men, are more likely to access available treatment [47]. Nevertheless, all the fit indices were excellent and ΔCFI, for defining invariance, was within the recommended cut-off [40, 41]. This result suggests that the unidimensional Swahili GAD-7 is invariant across both sex and age groups. Previous validation studies, from different regions, have also reported acceptable-to-excellent goodness-of-fit indices for the unidimensional structure of GAD-7 [44–46, 48] and its factorial invariance across both sex and age [44], but also across language-preference groups [49].

We examined convergent validity through correlation of scores from Swahili GAD-7 with those from PHQ-9 and the 12-item HIV stigma scale (measures of theoretically related constructs). We found good convergent validity of Swahili GAD-7 with these two measures. Our finding is consistent with findings from previous studies that investigated correlations of scores of GAD-7 with those of theoretically related construct measures such as measures of psychiatric morbidity [34, 49, 50], quality of life [45, 49] and disability [48].

We investigated discriminant validity by comparing mean GAD-7 scores of participants on first-line and second-line antiretroviral therapy. We used participant antiretroviral regimen as a proxy marker for perceived treatment success (those switching to second-line treatment perceiving poor treatment success expected to be related to higher GAD-7 scores). A change from first-line to second-line is a major step and is usually indicated because of toxicity or resistance to first-line treatment [51]. Whatever the case, there is an underlying treatment failure which may trigger anxiety symptoms when a patient is informed. Adjusting for sociodemographic and health factors, we found statistically significant group differences suggestive of good discriminant validity of Swahili GAD-7. García-Campayo, et al. [48] also found Spanish GAD-7 to have good discriminant validity while comparing mean scores of participants with a prior diagnosis of clinical GAD versus that of a control group.

### Study limitations

Despite the study strengths in terms of sample size and methodological approaches to evaluating reliability, factorial structure and validity of Swahili GAD-7, it is not without limitations. We recruited participants from one primary care centre, thus these findings may not be generalizable to other settings or regions. We used a proxy indicator—antiretroviral regimen—to examine discriminant validity. Future studies involving PLWHA should confirm our finding by investigating for group differences on GAD-7 scores using either or both standard biomarkers of disease progression (viral load and/or CD4 cell count). We did not explore the diagnostic accuracy of Swahili GAD-7, therefore we recommend a separate study in this or similar setting, exploring the sensitivity and specificity of this measure at different cut-off values, using a diverse sample of PLWHA, to identify the optimal cut-off score for identifying those in need of mental health treatment or referral services in HIV clinical settings.

## Conclusion

Our findings suggest that Swahili GAD-7 is a unidimensional scale with good psychometric properties among adults living with HIV from Kilifi, Kenya. It can be used to identify symptoms of GAD in similar research settings. However, to confidently identify those in need of mental health treatment or referral services in HIV primary care clinics, more research on the validity of Swahili GAD-7 is needed especially its discriminant validity and diagnostic accuracy at different cut-off scores. We recommend use of a diverse sample of PLWHA from this or similar settings.

## Data Availability

No additional data are available. Anyone interested in accessing the data reported in this article is free to write to the Data Governance Committee of the KEMRI-Wellcome Trust Research Programme who will review the application in ensuring that uses are compatible with the consent obtained from participants. Requests can be sent to the coordinator of the Data Governance Committee using the following email: dgc@kemri-wellcome.org.

## Abbreviations

ANCOVA: Analysis of covariance
CD4: Cluster of differentiation 4
CFA: Confirmatory factor analysis
CFI: Comparative fit index
ΔCFI: Difference in CFI
CGMR: Center for Geographic Medicine Research
GAD: Generalized anxiety disorder
GAD-7: 7-Item generalized anxiety disorder scale
ICC: Intra-class correlation coefficient
KMO: Kaiser–Meyer–Olkin
LMICs: Low- and middle-income countries
PHC: Primary Health Care
PHQ-9: 9-Item patient health questionnaire
PLWHA: Among people living with HIV/AIDS
RMSEA: Root mean square error of approximation
SCID: Statistical manual of mental disorders-IV
SSA: Sub-Saharan Africa
TLI: Tucker–Lewis index
WHO: World Health Organization
